# Free radical scavenging properties of pyrimidine derivatives

**DOI:** 10.1186/2191-2858-2-34

**Published:** 2012-11-14

**Authors:** Tabassum Bano, Nitin Kumar, Rupesh Dudhe

**Affiliations:** 1 Department of Pharmaceutical Technology, Meerut Institute of Engineering and Technology, Baghpat Bypass Crossing, NH-58, Delhi-Roorkee Highway, Meerut, Uttar Pradesh, 250005, India; 2 Department of Pharmacy, Galgotias University, Yamuna Expressway, Greater Noida, Uttar Pradesh, 201306, India

**Keywords:** Free radical, Antioxidant, Pyrimidine, Oxidative stress

## Abstract

Free radicals are well known for playing a dual role in our body- deleterious as well as beneficial. It includes a metabolic pathway for its generation. Oxidative stress in our body occurs due to excessive generation of free radicals and reduced level of antioxidants, but at low concentrations, these radicals help to perform normal physiological functions of the body. Scientific evidence suggests that antioxidants reduce the risk for chronic diseases including cancer and heart disease. This review shows current tendency in the pyrimidine synthesis and reveals the pyrimidine core to be a very potent moiety which can be a rich source for the synthesis of new compounds having desirable antioxidant activity.

## Review

### Introduction

Free radicals can be defined as the atoms, molecules, or ions with unpaired electrons in an open shell configuration. Sometimes, these free radicals may bear some charge, either positive, negative, or zero. They also play a significant role in combustion, atmospheric chemistry, polymerization, plasma chemistry, and many other chemical processes. Free radicals may generate different kinds of chemical and biological reactions in the body. Development of free radicals in the body is believed to involve in the development of various degenerative diseases; huge generation of free radicals particularly reactive oxygen species and their high activity may lead to progression of a number of pathological disturbances such as inflammation, atherosclerosis, cancer, Parkinson's disease, and Alzheimer's disease. This phenomenon of excessive production of free radicals is termed as oxidative stress. This oxidative stress has also been found to be implicated in many ailments such as heart disease and some age-related diseases. Thus, in a concise way, it can be said that excessive production of free radicals is harmful for the body.

**Figure 1:**
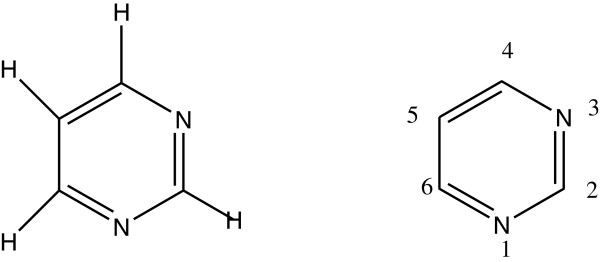
Basic structure of a pyrimidine ring.

Many heterocyclic compounds have been synthesized because of their wide range of biological activity [1]. Pyrimidine is a six-member heterocyclic compound that contains two nitrogen atoms at positions 1 and 3 (Figure 1). Pyrimidine derivatives have showed various biological activities such as antimicrobial, antitumor, antifungal, and antileishmanial activities and are also useful for the treatment of thyroid and leukemia [2]. In this article, the authors give an emphasis on antioxidant activity by pyrimidine-containing nucleus.

### Types of free radical reaction

There are three main types of free radical reactions:

1. Initiation reaction

2. Propagation reaction

3. Termination reaction [3]

### Sources of free radical generation

Various sources lead to the generation of free radicals:

• UV radiations, X-rays, gamma rays, and microwave radiation

• Reaction catalyzed by metals

• Oxygen free radicals in the atmosphere considered as pollutants

• Inflammation that initiates neutrophils and macrophages to produce reactive oxygen species and reactive nitrogen species

• In mitochondria-catalyzed electron transport reactions, oxygen free radicals produced as by-product

• Reactive oxygen species (ROS) generated by the metabolism of arachidonic acid, platelets, macrophages and smooth muscle cells

• Interaction with chemicals, automobile exhaust fumes, smoking of cigarettes and cigars

• Burning of organic matter during cooking, forest fires, volcanic activities

• Industrial effluents, excess chemicals, alcoholic intake, certain drugs, asbestos, certain pesticides and herbicides, some metal ions, fungal toxins, and xenobiotics

### Generation of free radical by metabolic pathway

Free radicals are mainly generated by various metabolic pathways such as lipid and peroxidation, gluconeogenesis, and glucuronidation. Generation of free radicals is first converted to hydrogen peroxide which is further reduced to water. This detoxification occurs during oxidative stress, where in the oxygen generates inside the body. The superoxide released by the oxidative phosphorylation pathway is the result of multiple enzymes; superoxide dismutase catalyzes the first step, and then various peroxides help in removing the hydrogen peroxide.

### Role of free radicals in our body

**Figure 2:**
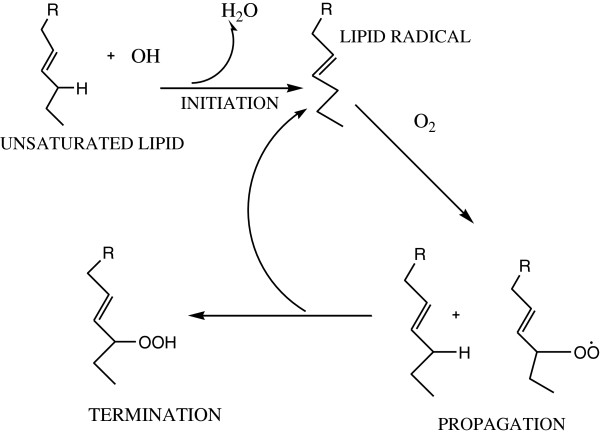
Biological pathway for the generation of free radical in living organisms.

Free radicals are naturally produced within the body (Figure 2), and their various effects are exhibited. The immune system is the main site for their attack. Generation of these free radicals decreases the effectiveness of the immune system. Free radicals also attack the cell membrane, resulting in the damage of a cell at such point that it must be discarded by the immune system. If the formation of free radicals and attacks are not controlled within the muscle during exercise, a large quantity of muscle could easily be damaged. Damaged muscles could in turn inhibit performance by the induction of fatigue [4,5].

### Diseases caused by the free radical generation in the human body

Oxygen is an element that is essential to life. Living systems have evolved to survive in the presence of molecular oxygen, which is also applicable to most biological systems. Oxidative properties of oxygen play a vital role in diverse biological systems. It includes the following:

• Neurodegenerative disorders such as Alzheimer's disease, Parkinson's disease, multiple sclerosis, amyotrophic lateral sclerosis, memory loss, and depression

• Cardiovascular diseases such as atherosclerosis, ischemic heart disease, cardiac hypertrophy, hypertension, shock, and trauma

• Pulmonary disorders such as inflammatory lung diseases such as asthma and chronic obstructive pulmonary disease

• Diseases associated with premature infants, including bronchopulmonary, dysplasia, periventricular leukomalacia, and intraventricular hemorrhage, retinopathy of prematurity, and necrotizing enterocolitis

• Autoimmune disease such as rheumatoid arthritis

• Renal disorders such as glomerulonephritis and tubulointerstitial nephritis, chronic renal failure, proteinuria, and uremia

• Gastrointestinal diseases such as peptic ulcer, inflammatory bowel disease, and colitis

• Tumors and cancer such as lung cancer, leukemia, and breast, ovary, and rectum cancers

• Eye diseases such as cataract and age-related retinal maculopathy. Aging process, diabetes, skin lesions, immunodepression, liver disease, pancreatitis, AIDS, infertility [6]

### General properties of antioxidants

The main characteristic of an antioxidant is its ability to trap free radicals. Scientific evidence suggests that antioxidants reduce the risk of chronic diseases including cancer and heart disease. Antioxidant activity was performed by DPPH free radical scavenging method using ascorbic acid as a standard drug.

### Classification of antioxidants

#### Natural antioxidants

**Figure 3:**
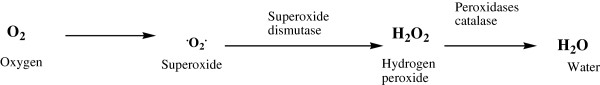
Enzymatic pathway for the detoxification of reactive oxygen species.

Antioxidants obtained from the natural source are known as natural antioxidants. They can be divided into following categories:

1. *Enzymes*. Enzyme such as superoxide dismutase, catalase, and glutathione peroxidase (GPx) attenuate the generation of reactive oxygen species by removing potential oxidants or by transferring ROS/RNS into relatively stable compounds (Figure 3). GPx reduces lipid peroxides (ROOH), formed by the oxidation of polyunsaturated fatty acids, to a stable nontoxic molecule - hydroxyl fatty acid (ROH).

2. *Lipid-soluble antioxidants*. This group of antioxidants is supposed to act as highly efficient scavengers, against lipid peroxyl radical, which is formed within the lipoprotein as a consequence of free radical chain reaction of lipid peroxidation.

3. *Water-soluble antioxidants*. These antioxidants cannot enter the lipid moiety of low density lipoprotein (LDL); these will be less efficient as these are principally unable to encounter most of these lyophilic radicals.

4. *Low molecular weight antioxidants*. These are subdivided into lipid-soluble antioxidants (tocopherol, carotenoids, quinones, bilirubin, and some polyphenols) and water-soluble antioxidants (ascorbic acid, uric acid, and polyphenols). These delay or inhibit cellular damage mainly through their free radical scavenging property.

#### Synthetic antioxidants

These are the compounds which are synthesized inside the laboratory. It includes butylated hydroxyl anisole, butylated hydroxy toluene, and tertiary butylated hydroxy quinone. These are most effective antioxidants and also come under the category of synthetic chemicals. ROS is a term which encompasses all highly reactive, oxygen-containing molecules, including free radicals. Some of the ROS along with their neutralizing antioxidants are given in Table 1[7-10].

**Table 1:** Various ROS and corresponding neutralizing antioxidants

**ROS**	**Neutralizing antioxidants**
Lipid peroxides	Beta-carotene, flavonoids, vitamin E, ubiquinone, glutathione1 peroxidase
Superoxide radical	Flavonoids, vitamin C, glutathione
Hydrogen peroxide	Vitamin E, vitamin C, glutathione, lipoic acid beta-carotene, flavonoids
Hydroxyl radical	Lipoic acid, vitamin C, glutathione, flavonoids

### Chemistry of the pyrimidine ring

**Figure 4:**
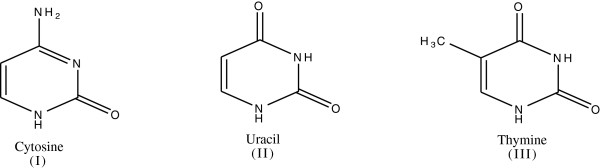
Basic unit of body building material.

The structure of the pyrimidine ring (Figure 4) is similar to benzene and pyridine. In a pyrimidine ring, as the number of nitrogen atoms increases the ring *π* electrons become less energetic; electrophilic aromatic substitution gets more difficult, while nucleophilic aromatic substitution resonance stabilization properties of pyrimidine may lead to the addition and ring cleavage reactions rather than substitutions.

### Leading compound as antioxidant templates

**Figure 5:**
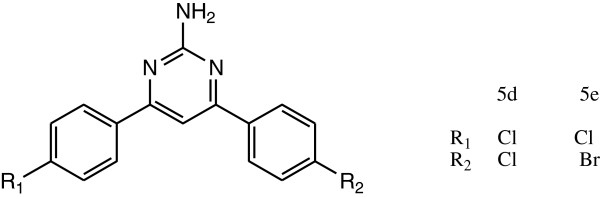
Compounds 5d and 5e.

Kumar et al. synthesized a novel series of 4,6-bisaryl-pyrimidin-2-amine derivative. All the synthesized compounds were evaluated for their antioxidant activity by nitric oxide and hydrogen peroxide free radical scavenging method using ascorbic acid as a standard drug. Among these synthesized derivatives, compound 5d by nitric oxide free radical scavenging method and compound 5e (Figure 5) by hydrogen peroxide free radical scavenging method show potent antioxidant activity as compare with the standard drug due to the presence of -Cl and -Br as the electron withdrawing group at positions R_1_and R_2_. IC_50_ values of the compounds are 0.019 and 0.020 mol/lit, respectively [11].

**Figure 6:**
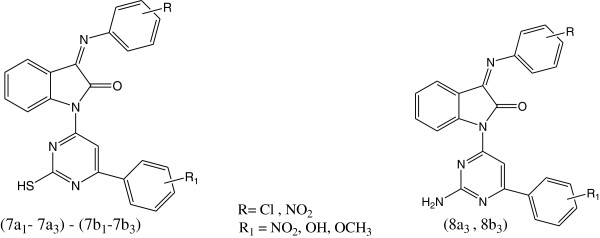
Substituted (Z)-3-(phenylimino)-1-(6-phenylpyrimidin-4-yl)indolin-2-one.

Mondal et al. synthesized a series of 1-(2-mercapto-6-(substituted phenyl)pyrimidine-4-yl)-3-(2-subsituted phenylimino) indoline-2-one and 1-(2-amino-6-(substituted phenyl)pyrimidine-4-yl)-3-(2-subsitutedphenylimino) indolin-2-one from different substituted chalconised indole-2,3-dione. Compounds 7a_2_, 7a_3_, 7b_2_, 7b_3_, 8a_3_, and 8b_3_ show more promising antioxidant activity due to the substitution of the SH and NH_2_ groups at the second position of the pyrimidine ring. (Figure 6) [12].

**Figure 7:**
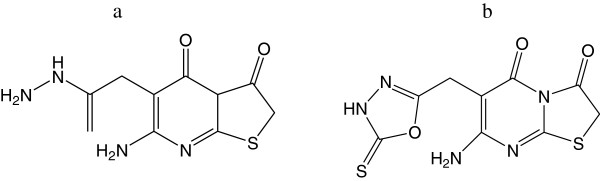
Thiazole and oxadiazole containing pyrimidine derivatives.

Abu-Hashem et al. synthesized a series of pyrimidine derivatives 6-amino-2-thiouracil with ethyl bromoacetate yielded ethyl 2-(7-amino-2,5-dioxo-3,5-dihydro-2 *H*-thiazolo[3,2-*a*pyrimidin-6-yl)acetate and 7-amino-6-[(5-thioxo-4,5-dihydro-1,3,4-oxadiazol-2-yl)-methyl]-5 *H*-thiazolo[3,2-*a*pyrimidine-3,5(2 *H*)-dione. The newly prepared compounds were subjected for their antioxidant activity; out of the synthesized compounds, compounds 7a and 7b (Figure 7) manifested potent antioxidative activity by lipid peroxidation assay. Novel thiazolopyrimidine derivatives incorporated with carbohydrazide, amino, oxadiazol, and other moieties which possess potential antioxidant activities [13].

**Figure 8:**
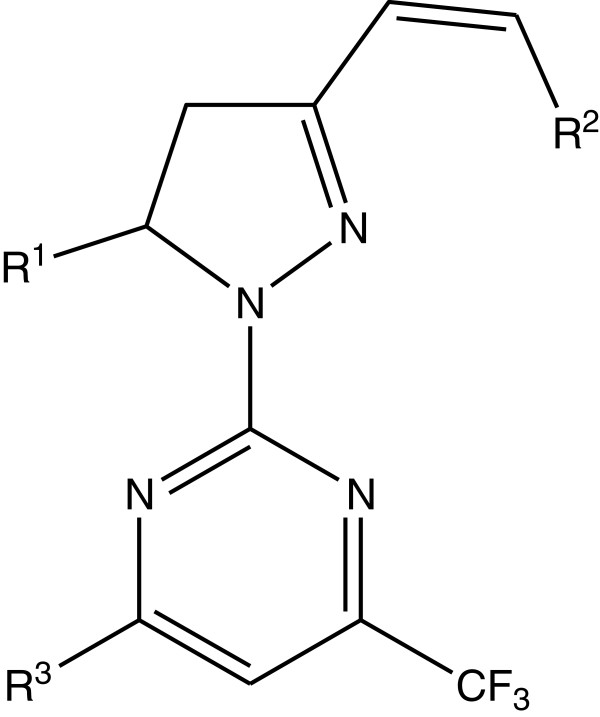
**2-(4,5-Dihydro-1** ***H*-pyrazol-1-yl)-pyrimidine.**

Gressler et al. synthesized a series of 4-trifluoromethyl-2-(5-aryl-3-styryl-1 *H*-pyrazol-1yl)-pyrimidine derivatives and were screened for their *in vitro* antioxidant activity. The antioxidant activity was evaluated using the DPPH and HRP/luminol/H_2_O_2_chemiluminescence assay method. The DPPH antioxidant assay measures the hydrogen-donating capacity of the molecules in the sample. On the other hand, the chemiluminescence method is based on the light emission produced by a chemical reaction. For the series 8a to 8e (Figure 8), resonance stabilization is not possible because the molecules do not have chain flexibility. Compound 8c shows potent antioxidant activity because of the presence of the SH group at R^3^ position [14].

**Figure 9:**
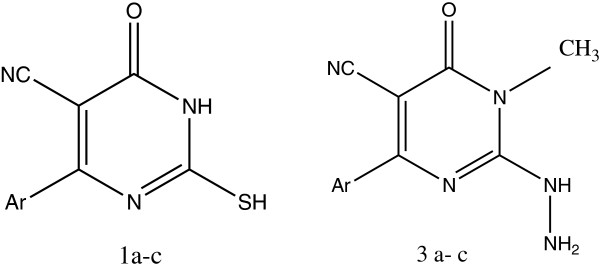
Structures of novel dihydropyrimidine carbonitrile and hydrazine derivative.

**Figure 10:**
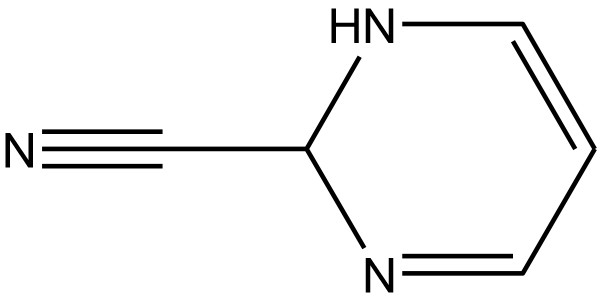
Structure of dihydropyrimidine carbonitrile.

Bhalgat et al. synthesized a series of novel dihydropyrimidine carbonitrile (compounds 1a to 1c), its dimethylated adduct (compounds 2a to 2c), and hydrazine derivative (compounds 3a to 3c); from compounds 2a to 2c, they also prepared its triazole fused derivatives (compounds 4a to 4c, 5a to 5c, and 6a to 6c). The tested compounds1a to 1c and 3a to 3c revealed potent antioxidant activity and showed the most promising results. This may be due to the presence of -NH and -SH groups in compounds1a to 1c and the presence of -NH-NH_2_ group in compounds 3a to 3c. The investigation of antioxidant screening revealed that some of the tested compounds particularly 1a, 1c, 3b, and 3c have shown more promising antioxidant activity as compare with the standard drug ascorbic acid, while other derivatives showed moderate activity (Figures 9 and 10) [15].

## Conclusions

The present review is concentrating on the synthesis and antioxidant activity of the pyrimidine nucleus. Pyrimidine is a unique molecule that is associated with several other biological activities. Among the antioxidants, it has the ability to trap the free radicals which are responsible for the generation of different diseases such as inflammation, skin lesions, immune depression, liver disease, pancreatitis, AIDS, infertility. Mainly antioxidants having pyrimidine nucleus are not able to enter in the lipid moiety of low density lipoprotein, so the penetration power of these types of compounds is very low, and they are least effective. To rectify this problem, the substitution on pyrimidine nucleus was made by different substitutions of Cl, Br, CF_3_, and NO_2_, which increased the penetration of molecules into the lipid membrane so that they increase the antioxidant activity by combining with the reactive oxygen species, which is generated by the different disease conditions. By making these changes on the pyrimidine nucleus, we are able to find out the most potent pyrimidine-substituted antioxidant compounds.

## Competing interests

The authors declare that they have no competing interests.

## References

[B1] Kumar SM Pavani M Bhalgat CM Deepthi R Mounika A Mudshinge SR Reas IJ Ghomi JS Ghasemzadeh MA Novel pyrimidine and its triazole fused derivatives: synthesis and investigation of antioxidant and anti-inflammatory activity J Serb Chem Soc 2011 76 679 684 10.2298/JSC100212057S

[B2] Gayathri G Nair BR Babu V Analysis of proximate and nutritional composition in the leaves of *Azima tetracantha* Lam Pharma Bio Sci 2011 2 1568 1570

[B3] March J Advanced organic chemistry reactions mechanism and structure 1985 3 Wiley, New York 165 179

[B4] Morrison RT Boyd RN Boyd RK Organic Chemistry 1992 6 Prentice Hall, Upper Saddle River 218 232

[B5] Acworth IN Bailey B Reactive oxygen species The handbook of oxidative metabolism 1997 ESA Inc, Chelmsford 89 95

[B6] Alessio HM Blasi ER Physical activity as a natural antioxidant booster and its effect on a healthy lifestyle Res Q Exerc Sport 1997 68 4 292 302 9421841 10.1080/02701367.1997.10608010

[B7] Saikat S Chakraborty R Sridhar C Reddy YS Biplab D Free radicals, antioxidants, diseases and phytomedicines: current status and future prospect Int J Ph Sci R 2010 3 1 91 100

[B8] Butkovica VL Bors KWJ Kinetic study of flavonoid reactions with stable radicals Agric Food Chem 2004 52 2816 2820 10.1021/jf049880h 15137819

[B9] Singh RP Sharad S Kapur S Free radicals and oxidative stress in neurodegenerative diseases, relevance of dietary antioxidants JIACM 2004 5 3 218 225

[B10] Lonita P Plasma polymerisation: study and application Chem Pap 2005 59 11 16

[B11] Kumar SM Pavani M Bhalgat CM Deepthi R Mounika A Mudshinge SR In-vitro antioxidant studies of 4,6-bis aryl-pyrimidin- 2-amine derivatives Inter JR Ph Bio Sci 2009 2 1568 1570

[B12] Mondal P Jana S Kanthal LK Synthesis of novel mercapto-pyrimidine and amino-pyrimidine derivatives of indoline-2-one as potential antioxidant & antibacterial agent T Ph Res 2010 3 17 26

[B13] Abu-Hashem AA Youssef MM Hoda AR Synthesis, antioxidant, antituomer activities of some new thiazolopyrimidines, pyrrolothiazolopyrimidines and triazolopyrrolothiazolopyrimidines derivatives J Chi Chem Soc 2011 58 41 48 10.1002/jccs.201190056

[B14] Gressler V Moura S Flores AFC Flores DC Colepicolo P Pinto E Antioxidant and antimicrobial properties of 2-(4,5-dihydro-1H-pyrazol-1-yl)- pyrimidine and 1-carboxamidino-1H-pyrazole derivatives J Braz Chem Soc 2010 21 1 7 10.1590/S0103-50532010000100001

[B15] Bhalgat CM Ali MI Arsab GR In-vitro antioxidant studies of 4,6-bis aryl-pyrimidin- 2-amine derivatives J Chem 2011 (in press)

